# Aggregation affects optical properties and photothermal heating of gold nanospheres

**DOI:** 10.1038/s41598-020-79393-w

**Published:** 2021-01-13

**Authors:** Yiru Wang, Zhe Gao, Zonghu Han, Yilin Liu, Huan Yang, Taner Akkin, Christopher J. Hogan, John C. Bischof

**Affiliations:** 1 Department of Mechanical Engineering, University of Minnesota, Twin Cities, Minneapolis, MN 55455 USA; 2 Department of Biomedical Engineering, University of Minnesota, Twin Cities, Minneapolis, MN 55455 USA

**Keywords:** Nanoscience and technology, Optics and photonics

## Abstract

Laser heating of gold nanospheres (GNS) is increasingly prevalent in biomedical applications due to tunable optical properties that determine heating efficiency. Although many geometric parameters (i.e. size, morphology) can affect optical properties of individual GNS and their heating, no specific studies of how GNS aggregation affects heating have been carried out. We posit here that aggregation, which can occur within some biological systems, will significantly impact the optical and therefore heating properties of GNS. To address this, we employed discrete dipole approximation (DDA) simulations, Ultraviolet–Visible spectroscopy (UV–Vis) and laser calorimetry on GNS primary particles with diameters (5, 16, 30 nm) and their aggregates that contain 2 to 30 GNS particles. DDA shows that aggregation can reduce the extinction cross-section on a per particle basis by 17–28%. Experimental measurement by UV–Vis and laser calorimetry on aggregates also show up to a 25% reduction in extinction coefficient and significantly lower heating (~ 10%) compared to dispersed GNS. In addition, comparison of select aggregates shows even larger extinction cross section drops in sparse vs. dense aggregates. This work shows that GNS aggregation can change optical properties and reduce heating and provides a new framework for exploring this effect during laser heating of nanomaterial solutions.

## Introduction

The laser heating of gold nanoparticles (GNS) is increasingly applied for the controlled heating of biological systems, with applications ranging in scale from the molecular to bulk tissue^1–3^. The key advantage of laser GNS heating is extremely efficient light-to-heat (photon energy-to-thermal energy) conversion and consequently, extremely rapid heating rates. For reference, iron oxide nanoparticles have been used for decades in cancer treatments, and typically have specific absorption rates in the 100’s of W/mg Fe; whereas laser heating of GNS can yield specific absorption rates that are orders of magnitude higher on a per weight bases: 100’s W/µg Au^1,4^. In addition, GNS surface chemistry is well-developed, enabling tailored coating and functionalization for biological applications^5,6^. Beyond traditional plasmonic tumor ablation^7^, there is growing interest for GNS and gold nanorods (GNR) photothermal heating in other aspects in biomedicine (see Table 1). Ultimately, all of these applications rely on GNS to convert laser light energy to heat via surface plasmon resonance^8^. Ideally, this heat for a single GNS can be simply estimated from the absorption cross section of a single GNS multiplied by the laser fluence rate (Q_GNS_ = I C_abs_), with the optical properties of the GNS established. Conventional tools to study GNS photothermal conversion include computational approaches (e.g. discrete dipole approximation and finite difference methods), optical property measurements (e.g. UV–Vis spectrometry), and GNS suspension photothermal conversion “calorimetry” experiments (e.g. in a droplet, in a cuvette in vacuum or standard room temperature and pressure)^9–14^.

**Table 1:** Emerging biomedical applications of laser GNS heating.

Application	Gold nanoparticle	Laser
Diagnostic assays^49,50^	30–100 nm GNS	CW, 532 nm
Cryopreserved biomaterial Re-warming^35,51^	1064 nm resonance GNR	ms pulsed, 1064 nm
Controlled drug release^52,53^	2–5 nm GNS	250 or 514 nm
	25 nm GNR	656 or 808 nm
	44–60 nm nanostars	803 or 850 nm
Tissue stimulation^54^	Cardiac, 200 nm GNS	ps pulsed, 532 nm
	Neuron, 750–810 nm GNR	CW, 780 nm and pulsed 800 nm
	Skin, 8–10 nm GNS	CW, 532 nm
Antibacterial biofilm treatment^55–57^	10–15, 40 nm GNS	CW, 665 nm and ns pulsed and 420–570 nm
	785 nm resonance GNR	CW, 785 nm
Selective virus inactivation^58^	805 nm resonance GNR	fs pulsed, 805 nm
Selective protein inactivation^59^	15, 30 nm GNS	ns pulsed, 532 nm

However, caution must be exercised when extrapolating measurements of GNS photothermal conversion to performance under idealized conditions to real biological systems. During application, the distribution of GNS can be non-uniform and higher ionic strength and protein concentration in biological systems can lead to GNS aggregation^15–18^. Recent work^9^ reveals that the polydispersity of the primary GNS diameters alone can result in heating that is 70% lower than theoretical predictions. Similar to polydispersity, aggregation has the potential to further influence GNS photothermal conversion. Aggregation has been shown to directly affect both the optical properties of particles in other systems, and heating by other mechanisms. For example, aggregated aerosol particles, most notably, black carbon soot, have distinct photothermal behavior from spherical particles^19^. Highly aggregated iron oxide nanoparticles have reduced specific absorption rates in radiofrequency magnetic fields, in comparison to unaggregated particles^18,20^. There are additionally computational studies showing GNS optical extinction spectrum shift under aggregation^21–23^.

While the influences of many geometric parameters on optical properties of GNS have been studied, including aggregate geometry (e.g. GNS dimers, GNS chains, GNS coated hollow spheres and cubes by eight GNS), gap distance in assembly, GNS distribution pattern, incident light polarization, and incident light angle, to the best of our knowledge, no specific studies of how GNS aggregation will affect heating, arguably the most important outcome in biothermal applications^8,24,25^, have yet been undertaken. In an effort to begin to understand this, we study here the influence of GNS aggregation on laser GNS heating both computationally and experimentally. We chose a typical size range of GNS (5, 16 and 30 nm) for photothermal conversion and early-stage aggregate sizes (2 to 30 primary spheres particles) for these studies. In computational work aggregates are modelled as relatively dense statistically fractal assemblies with fractal (Hausdorff) dimensions of 2.4. The discrete dipole approximation (DDA) is utilized to examine how aggregation changes GNS optical properties. These calculations are then compared to cuvette “laser GNS calorimetry” and extinction cross section measurements by UV–Vis.

## Methods

### Calculation methods

#### Geometry generation

The earliest stages of aggregation proceed through monomer (primary particle) addition to a growing aggregate, as the concentration of monomers greatly exceeds the aggregate concentration. Both diffusion-limited and reaction-limited aggregation mechanisms suggest that such early-stage “cluster-monomer” formed aggregates are relatively dense^26^. Here, we attempt to examine the influence of the aggregation size and the geometry (dense vs sparse) on the photothermal heating. For this purpose, it is convenient to describe aggregate morphology as statistically fractal, with each aggregate approximately obeying the scaling law^27,28^:

1 $$ {\mathbf{N}} = {\mathbf{k}}_{{\varvec{f}}} \left( {\frac{{{\varvec{R}}_{{\varvec{g}}} }}{{\varvec{a}}}} \right)^{{{\varvec{D}}_{{\varvec{f}}} }} , $$

where N is the number of primary particles in the aggregate, ***a*** is the radii of the primary particle, ${\varvec{k}}_{{\varvec{f}}}$ is the fractal prefactor, ${\varvec{D}}_{{\varvec{f}}}$ is the fractal dimension. All GNS aggregate geometries were generated using a Sequential Algorithm (SA) described previously^29,30^. Though the SA approach has difficultly reproducing the features of large (N > 100) aggregates, it is a robust algorithm for smaller aggregates as in this study, and is capable of generating both sparse and dense aggregates. The SA enables random generation of an aggregate with prescribed $k_{f}$ and $D_{f}$; identical primary particles are added to the aggregate one by one, satisfying Eq. (1) at each step. In the present study, we set $k_{f} = 1.6$, and the number of the primary particles in the aggregates as 5, 10, 15, 20, 25, or 30. To study whether aggregate morphology had any influence, the fractal dimension was adjusted to control the assembly state (dense or sparse) with $D_{f} =$ 2.4 (dense), 1.4 (sparse).

#### Discrete dipole approximation calculation

The DDA is a solution approximation for Maxwell’s equations involving discretization of the target, either regularly or irregularly shaped, into a finite array of dipoles. DDSCAT 7.3 was used to perform DDA simulations^31^. Validation of the parameter configuration was made by matching our results for a single 30 nm GNS to previous work^9^. We set 4 dipoles/nm, which provided extra accuracy while still being computationally accessible (up to 2–3 weeks for one case). To mimic the most common photothermal conversion application, an unpolarized single wavelength light source was used and water was chosen as the surrounding medium. Although the isotropy of isolated GNS makes the optical properties independent of incident light direction, GNS aggregation is usually anisotropic, thus requiring an average of the calculation results by several orientations. After trying various orientations, we ultimately settled on 5 to be a sufficient number because adding further orientations has minimal impact on the outcome (Fig. S1). Table 2 shows the calculation matrix in this study. We did a calculation with coarse wavelength step sizes to find the overall shape of spectrum curves. We then refined the step size close to the plasmonic peaks for accuracy. In order to compare the photothermal conversion of various size GNS aggregates, the optical properties (i.e. cross-sections) were averaged by GNS numbers in an aggregation cluster with the equation:

2 $$ {\varvec{C}}_{{{\varvec{ext}},{\varvec{ave}}}} = \frac{{{\varvec{Q}}_{{{\varvec{abs}}}} {\varvec{A}}_{{{\varvec{eff}}}} }}{{{\varvec{N}}_{{{\varvec{agg}}}} }}, $$

3 $$ {\varvec{C}}_{{{\varvec{abs}},{\varvec{ave}}}} = \frac{{{\varvec{Q}}_{{{\varvec{abs}}}} {\varvec{A}}_{{{\varvec{eff}}}} }}{{{\varvec{N}}_{{{\varvec{agg}}}} }}, $$

4 $$ {\varvec{C}}_{{{\varvec{sca}},{\varvec{ave}}}} = \frac{{{\varvec{Q}}_{{{\varvec{sca}}}} {\varvec{A}}_{{{\varvec{eff}}}} }}{{{\varvec{N}}_{{{\varvec{agg}}}} }}, $$

where $$C_{ext,ave} , C_{abs,ave} , $$ and $$C_{sca,ave}$$ are the number-averaged extinction, absorption, and scattering cross-sections of GNS in an aggregation cluster; Q_ext_, Q_abs_ and Q_sca_ are the extinction, absorption and scattering efficiency factors of the whole cluster and the direct output by DDSCAT; A_eff_ is the effective geometric cross-section area; and N_agg_ is the number of GNS in the aggregate. The numerators are the extinction, absorption, scattering cross-sections of the aggregation parcel.

**Table 2:** DDA simulation matrix of aggregated GNS.

Parameters	Values
GNS aggregate size	2, 3, 4, 5, 10, 20, 30
GNS diameter (nm)	5, 16, 30
Wavelength (nm)	450–950
Wavelength step size (nm)	20 for bulk; 4 for range of resonance peak (± 50)

#### Converting optical properties into heating

In order to provide a direct comparison between calculation results and experimental data, we used the theoretical per-particle optical properties to predict the heat generation rate (Q) of the GNS solutions in the experimental conditions. The bulk solutions’ optical properties are predicted as:

5 $$ \mu_{t} = C_{ext,ave} \times N, $$

6 $$ \mu_{a} = C_{abs,ave} \times N, $$

7 $$ \mu_{s} = C_{sca,ave} \times N, $$

where µ_t,_ µ_a_, and µ_s_ are bulk solution’s extinction, absorption, and scattering coefficients, respectively; and N is the experimentally estimated concentration of the GNS in the laser calorimetry experiment. The predicted heat generation of the GNS solution in the cuvette can be calculated as

8 $$ {\varvec{Q}} = {\varvec{P}}_{{{\varvec{loss}}}} \times {\varvec{\eta}} = \user2{ P}_{{{\varvec{loss}}}} \times \frac{{{\varvec{C}}_{{{\varvec{abs}},{\varvec{aver}}}} }}{{{\varvec{C}}_{{{\varvec{ext}},{\varvec{ave}}}} }}, $$

where ${\varvec{P}}_{{{\varvec{loss}}}}$ is the predicted laser power loss through the GNS solutions in the cuvette. It is calculated by

9 $$ {\varvec{P}}_{{{\varvec{loss}}}} = {\varvec{P}}_{{{\varvec{in}}}} - {\varvec{P}}_{{{\varvec{out}}}} = \left( {1 - \exp \left( { - {\varvec{\mu}}_{{\varvec{t}}} \times {\varvec{L}}} \right)} \right) \times {\varvec{P}}_{{{\varvec{in}}}} , $$

where P_in_ is the experimental inlet laser power and L is the light path length through the solution (1 cm). In short, according to the DDA predicted photothermal efficiency (η) and other parameters, we can calculate the predicted but still experimentally informed heat generation (Q) of the 1 mL GNS solution, mimicking the laser calorimetry experiment.

### Experimental methods

#### GNS synthesis and characterization

GNS were synthesized following published methods^32,33^. All glassware and stir bars were cleaned by Aqua Regia prior to synthesis. All chemicals were purchased from Sigma-Aldrich and used without further purification. The 16 nm GNS were produced by adding 0.5 mL 3% (W/V) sodium citrate into boiled 50 mL 0.25 mM HAuCl_4_ under vigorous stirring. The resulting 16 nm GNS were used both for aggregate study and as seeds for 30 nm GNS. The 30 nm GNS were synthesized by adding 0.875 mL 25 mM HAuCl_4_, 15 mM sodium citrate, 12.5 mL as-made 16 nm GNS and 25 mM hydroquinone successively into vigorously stirred MilliQ water with continued stirring for 30 min at room temperature. The resulting GNS were characterized by dynamic light scattering (DLS, Brookhaven Zeta PALS instrument), transmission electron microscopy (TEM, FEI Tecnai T12), and UV–Vis spectroscopy (Ocean Optics, Dunedin). We also synthesized 5.5 nm CTAB capped GNS following a previously published protocol^34^. This synthesis method requires excess amount of CTAB to stabilize GNS and the CTAB precipitate affects DLS reading. In addition, upon purification irreversible aggregation of 5.5 nm GNS occurred making further experiments impossible.

#### Aggregate induction and stabilization

16 nm GNS aggregation was induced by modifying a previously published method^16^. A series of 50 mL NaCl + transferrin (TF) stock solutions were made as follows: 25NaCl + TF: 0.5844 g NaCl + 0.004 g TF; 100NaCl + TF: 2.3376 g NaCl + 0.004 g TF; 400NaCl + TF: 9.3504 g NaCl + 0.004 g TF. The number on the NaCl + TF stock solution indicated the final concentration of NaCl in the aggregate suspension. For example: 25NaCl + TF resulted in 25 mM NaCl in the final GNS solution. A TF stock solution was made by dissolving 0.0064 g TF in 20 mL MilliQ water. To induce the aggregation, 0.4 mL NaCl + TF stock solution was added to 2.4 mL GNS solution and vigorously vortexed for 1 min, then 0.4 mL TF stock solution was added to stabilize the aggregates.

TF was not sufficient to stabilize 30 nm GNS aggregates, and we observed further aggregation after TF was added. Therefore, we used polyvinyl pyrrolidine (PVP10, MW = 10,000 g/mol) to stabilize 30 nm GNS aggregates induced by NaCl addition. The protocol was optimized by both varying NaCl and PVP additions to generate small aggregates. Similar to 16 nm GNS, a series 10 mL NaCl stock solutions were made: 100 NaCl: 0.46752 g NaCl; 200 NaCl: 0.93504 g NaCl; 400 NaCl: 1.87008 g NaCl. A PVP10 stock solution was made by dissolving 25 mg PVP10 in 10 mL H_2_O. The aggregation was induced by adding 0.4 mL stock NaCl to 2.4 mL GNS solution and vigorously vortexed for 1 min, then 0.4 mL PVP10 stock solution was added to stop further aggregation. Once formed, the aggregates were characterized by DLS, UV–Vis and TEM as described in GNS synthesis.

#### GNS laser calorimetry measurement

The laser calorimetry system has been used previously to assess laser heating of a series of GNS, GNR, and gold nanoshell solutions^9,35,36^. Freshly prepared GNS suspensions are loaded into a four-side plastic optical cuvette and heated with a 532 nm CW laser (190 mW). To minimize measurement errors, four temperature thermocouples and a magnetic stirrer were used. The GNS suspension was photoheated to steady state (< 0.1 °C temperature change in 1 min) and a maximum temperature rise was measured (∆T, °C). A resistive heater with known power was placed in the cuvette system and used to calibrate the specific absorption rate (SAR) assuming the laser GNS heat source of the same power produced the same temperature rise. Resistive heater calibration was performed multiple times over a wide power range (25–250 mW). Calibrated in a previous study^9^, there was a linear correlation between ∆T and the experimental photothermal heat generation (P_exp_, mW) with a fitting coefficient (Eq. (10)):

10 $$ {\text{SAR}} = P_{\exp } = 16.855\Delta T $$

Cuvette calorimetry measurements were conducted three times for each GNS aggregate suspension under the same conditions.

### Statistical analysis

The error bars are standard deviations. The one-way analysis of variance (ANOVA) with Tukey’s multiple-comparison tests (GraphPad Prism, GraphPad Software Inc.) was performed on photothermal heat data analysis. Statistical significance compared with fresh samples is indicated with asterisks: *p < 0.05; **p < 0.01.

## Results and discussion

### Calculation optical results

This work seeks to answer the question of whether optical property changes due to GNS aggregation will change their heating properties for GNS biomedical applications. DDA numerical simulations of optical properties were performed on a large set of GNS of various sizes (5, 16 and 30 nm) and aggregation states (2–30 particles). Very compact geometries of GNS aggregation with direct particle contact were numerically calculated to represent the extreme case where the interparticle effect was maximized. For other GNS aggregate geometries, the optical properties should fall between isolated GNS and our extreme case.

**Figure 1:**
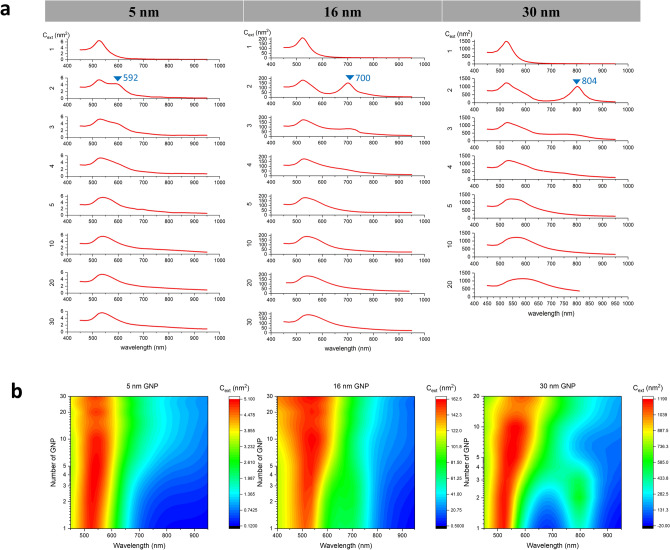
Averaged extinction cross-section of aggregated 5, 16, and 30 nm GNP by spectra plots (**a**) and contour plots produced with fitting and smoothing algorithms (**b**). (**a**) Number on the left of the y-axis indicate the number of GNPs in the aggregate. A second peak can be observed for 2 particle aggregations at various wavelength and gradually diminished with increase of aggregation sizes up to 30 (5 and 16 nm GNP) and up to 20 (30 nm GNP).

**Figure 2:**
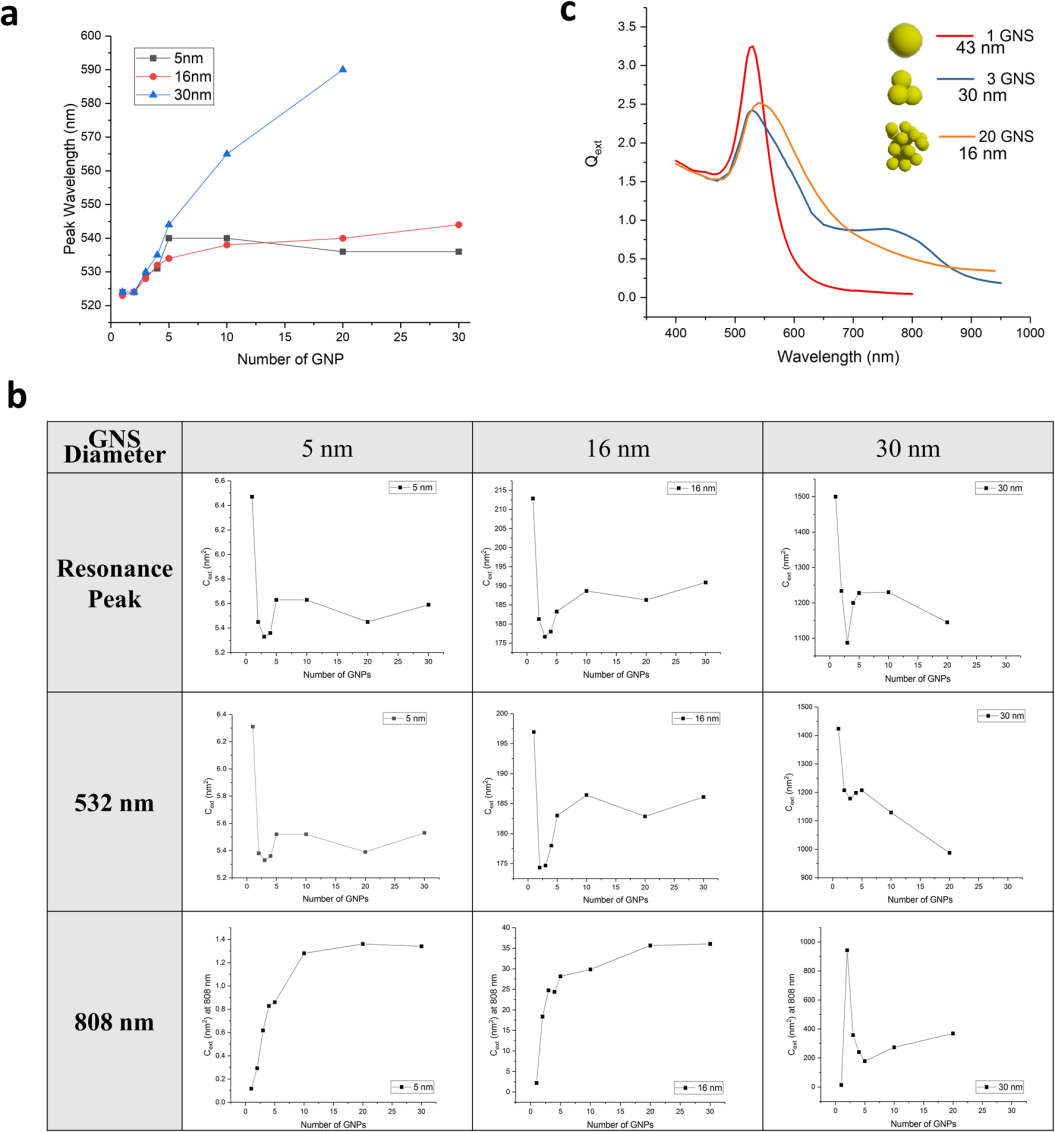
(**a**) Plasmon resonance peak of aggregated GNP. The larger the aggregation size and the larger the GNP diameter, the more red shifted the aggregated cluster will be. (**b**) Averaged per-particle extinction cross-section plots of 5, 16, and 30 nm diameter GNP aggregations at the resonance peak (532 nm (green laser heating) and 808 nm (NIR laser heating)). (**c**) To explore the way to generate the most heat by the same amount of gold, the extinction coefficient (Q_ext_) of 1, 3, and 20 particle clusters with very close effective radius (< 1% difference) are compared. We can see the single GNP has the highest extinction at resonance peak, while an aggregation cluster with larger number of GNP is slightly better than the small cluster.

For smaller aggregates (N ≤ 5), we assume idealized close-packed assemblies (no gaps between particles, geometries shown in Table 3). The gap distance effect is not considered in our model. We expected that the aggregation effect will be more prominent for dense, compact geometries due to strong overlapping and coupling of the electro-magnetic fields from adjacent particles, while GNS will behave as isolated particles when separated by sufficient distance. Thus, we assume each adjacent GNS is single point contacted with its neighbour(s). The optical response of 5, 16, and 30 nm diameter GNS were chosen because (1) they are within a typical size range and fabrication methods are well-established, and (2) they cover a relatively large size range within the computing capacity tolerance (up to 2–3 weeks’ computation for one case). The DDA results for adsorption and scattering cross-sections for every case (1–30 GNS of 5, 16 nm and 1–20 GNS of 30 nm) are shown in Fig. S2. The $$C_{abs}$$ is much larger than the $$C_{sca}$$ (difference:$${ }\sim 10{ }\;{\text{to }}\; > \;{ }100{\text{-fold}}$$) in most cases, indicating the $$C_{ext}$$ ($$= C_{abs} + C_{sca}$$) is dominated and close to $$C_{abs}$$. Therefore, we took the predicted extinction cross-sections as the representative parameter to study the trend of optical property changes with aggregation (Fig. 1). The primary plasmonic peak showed broadening and red shifting for all three sizes of GNS, while larger GNS (30 nm) showed more prominent changes (Fig. 2a). A prominent secondary plasmon resonance peak is observed for aggregates composed of 2–4 primary GNS, as shown in Fig. 1. As GNS diameters increased, the secondary peak was red-shifted and diminishes in larger aggregates (> 4 GNS). This secondary peak is in some sense similar to the NIR peaks from GNRs due to the longitudinal plasmon resonances. At the resonance peak, the extinction cross-section drops significantly (17–28%) and gradually recovers (partially) with aggregate growth as shown in Fig. 2b. This could be induced by the “shape effect” at smaller aggregate sizes, especially for dimers and trimers. However, GNS diameter plays a role as well; the resonance peak location for 5 nm GNS is relatively insensitive to aggregate size, for peak location for 16 nm GNS increases slightly with increasing aggregate size, and the peak for 30 nm GNS decreases with increasing aggregate size. During laser heating applications, a constant laser wavelength is usually applied instead of matching the GNS resonance peak (Fig. 2b). For instance, 532 and 808 nm are commonly used photothermal laser wavelengths in biomedicine. The optical extinction of GNS aggregates at the irradiated wavelength is hence not necessarily the extinction cross section at the resonance peak, as the peak’s location depends on both the particle size and the aggregate size. By comparing $$C_{ext}$$ at peak wavelength and at 532 nm, it suggests that when excited at 532 nm, the $$C_{ext} $$ of 5 nm GNS are very close to that at resonance peak; the $$C_{ext}$$ of 16 nm GNS and their aggregates are slightly lower than those at resonance peak; and the $$C_{ext}$$ of 30 nm GNS keeps decreasing with aggregate size. At 808 nm, the extinction cross-section increases rapidly during aggregation for all GNS diameters. The spike of 30 nm GNS at small aggregation sizes is caused by the secondary resonance peak (804 nm).

**Table 3:**
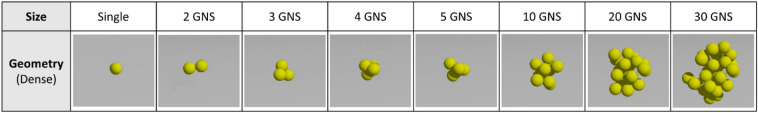
Dense GNS aggregation geometries.

Though differences were observed for different sizes of GNS, all the aggregates in our DDA simulations (2–30 GNS) show a rapid decrease and then partial recovery of optical extinction (reflective of heat generation in bulk) with increase in aggregate size. The GNS trimer aggregates of all particle sizes has the lowest average optical extinction at the plasmon resonance peak. Then the trimers are followed by dimers of all particles sizes as compared to all other aggregation states. Based on the Maxwell Garnett effect, the electromagnetic coupling between GNS in colloidal solution becomes increasingly important when the center-to-center distance is smaller than 5 times the particle radius^37,38^. Besides the surface plasmon resonance of single isolated GNS, the inter-particle dipole coupling together induced the unique extinction spectra of an aggregation cluster. The cluster geometry of smaller aggregation clusters is more regular; therefore, the inter-particle resonance is more anisotropic by the non-uniform electron distribution^37^. This effect is supposed to count for the lowest primary extinction peak for dimers and the rapid secondary peak. On the contrary, the collective effect is spread more equally to the whole spectra for larger clusters, thus showing some recovery at the primary peak comparing to smaller clusters. Despite the aggregate size, different-sized GNS responded to aggregation differently. Interestingly, 30 nm GNS had a more prominent resonance peak shift than smaller ones. This peak shift results in a rapid visible color change which can also be used to assess degree of aggregation in colorimetric assays^39,40^.

To explore how to use gold more efficiently in photothermal heating, we assumed we have fixed total mass of gold. We then compared the heating of a certain gold mass in the shape of a single large GNS, three particle aggregates, and 20 particle aggregates in Fig. 2c. Since the total gold mass of the three geometries are same (< 1% difference) for the three cases, the extinction efficiency factor (Q_ext_) is reflective of the heating ability. We found the Q_ext_ for the single particle to be far higher than that of aggregate clusters. In summary, modeling predicts that a single larger particle is more effective than the same mass of smaller aggregated particles for heating applications GNS.

### Experimental results

**Figure 3:**
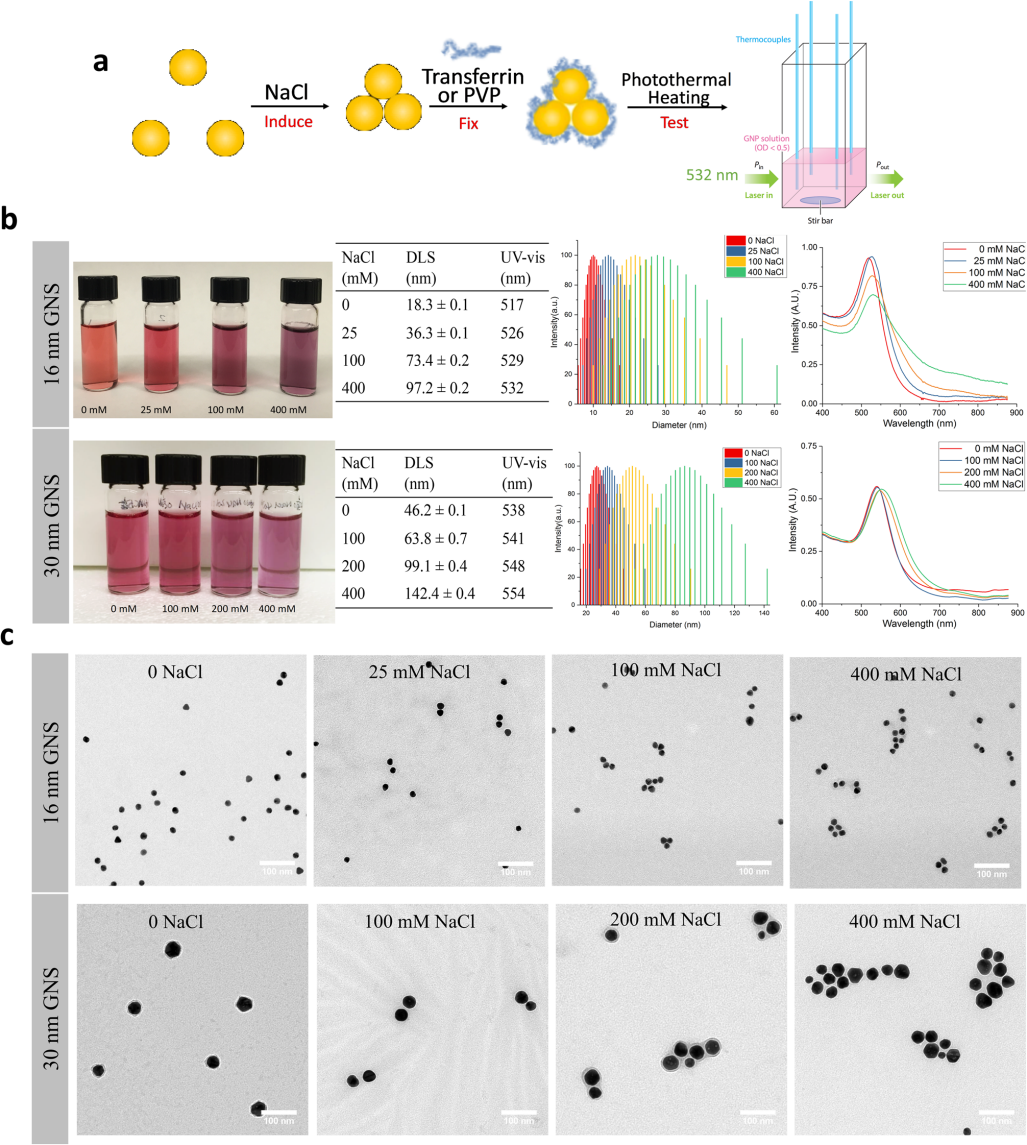
GNP aggregation photothermal conversion experiment preparation. (**a**) Experiment scheme: induce and fix controlled-size GNP aggregation in solution and test by cuvette settings. (**b**) Visual picture, DLS and UV–Vis characterization of GNP aggregation solutions. (**c**) TEM Characterization of GNP aggregation solution samples in (**b**), 100 nm scale bar.

Photothermal conversion experiments were conducted to compare to our numerical results. The scheme of GNS aggregation induction, stabilization and photothermal heating is shown in Fig. 3. We selected 16 and 30 nm GNS for the experiment as we were unable to prepare discrete 5 nm GNS that are colloidal stable in water and enable the creation of stable fixed number GNS aggregates as described in GNS synthesis and characterization section. Citrate coated GNS instantly aggregate in salt solutions due to the neutralization of surface charge^16,41^. The size of irreversible aggregation will keep increasing and eventually sediment out of the solution. Sediments impede the quantitative comparison of the heating^42^. Although DNA linking is another widely applied method to study GNS aggregation for large-size, customized uniform aggregation shapes, it has a major drawback of poor thermal stability^43^. To prohibit further aggregate growth via salt-induced aggregation, a stabilizer was added to the GNS solution 1 min after NaCl addition. Transferrin was used to fix 16 nm GNS aggregates followed by a published protocol^16^. However, transferrin was not able to stabilize GNS aggregates formed by 30 nm GNS. Polyvinyl pyrrolidone (PVP) has strong affinity to wrap around nanostructures regardless of surface charge, size and material^44,45^. Herein, PVP was used to stabilize aggregates formed by 30 nm GNS. The GNS aggregate samples and controls were characterized by DLS, UV–Vis and TEM (Fig. 3b,c). Different sized GNS require different amount of NaCl to induce aggregation. It was observed that 25 mM NaCl did not induce 30 nm GNS aggregation. No hydrodynamic size nor extinction peak change was observed in 30 nm GNS with 25 mM NaCl addition. The color change and size distribution of 16 nm GNS were very similar to those of a previous GNS aggregation study^16^, indicating good replication of 16 nm GNS aggregation formation. PVP stabilized NaCl induced 30 nm GNS aggregation was assessed by DLS (Fig. 3b). Both transferrin and PVP stabilized GNS aggregates showed good colloidal stability over 2 days without detectable hydrodynamic diameter changes (Fig. S3), so that we assume the aggregation state was the same when performing heat conversion test. Moreover, the red shift of extinction peak in the UV–Vis spectra also indicates successful induction of aggregation. Figures 3 and S4 show representative TEM images of GNS aggregates. The aggregate size, geometry as well as the gap between adjacent GNS are not uniform. Although we cannot precisely control the aggregation induced by NaCl addition, a clear trend of growing aggregation size was confirmed by TEM imaging (Fig. 3c).

From the UV–Vis curves (Fig. 3b), there is an up to 25% drop in the peak absorbance (i.e. extinction coefficient) for the 16 nm GNS aggregates when adding 400 mM NaCl into the as-synthesized GNS, though this trend is less apparent for the larger (30 nm) GNS. The drop in the extinction coefficient indicates the reduced interaction between the GNS with the incident light, highly suggesting a reduced heat generation by GNS aggregate during laser excitation.

**Figure 4:**
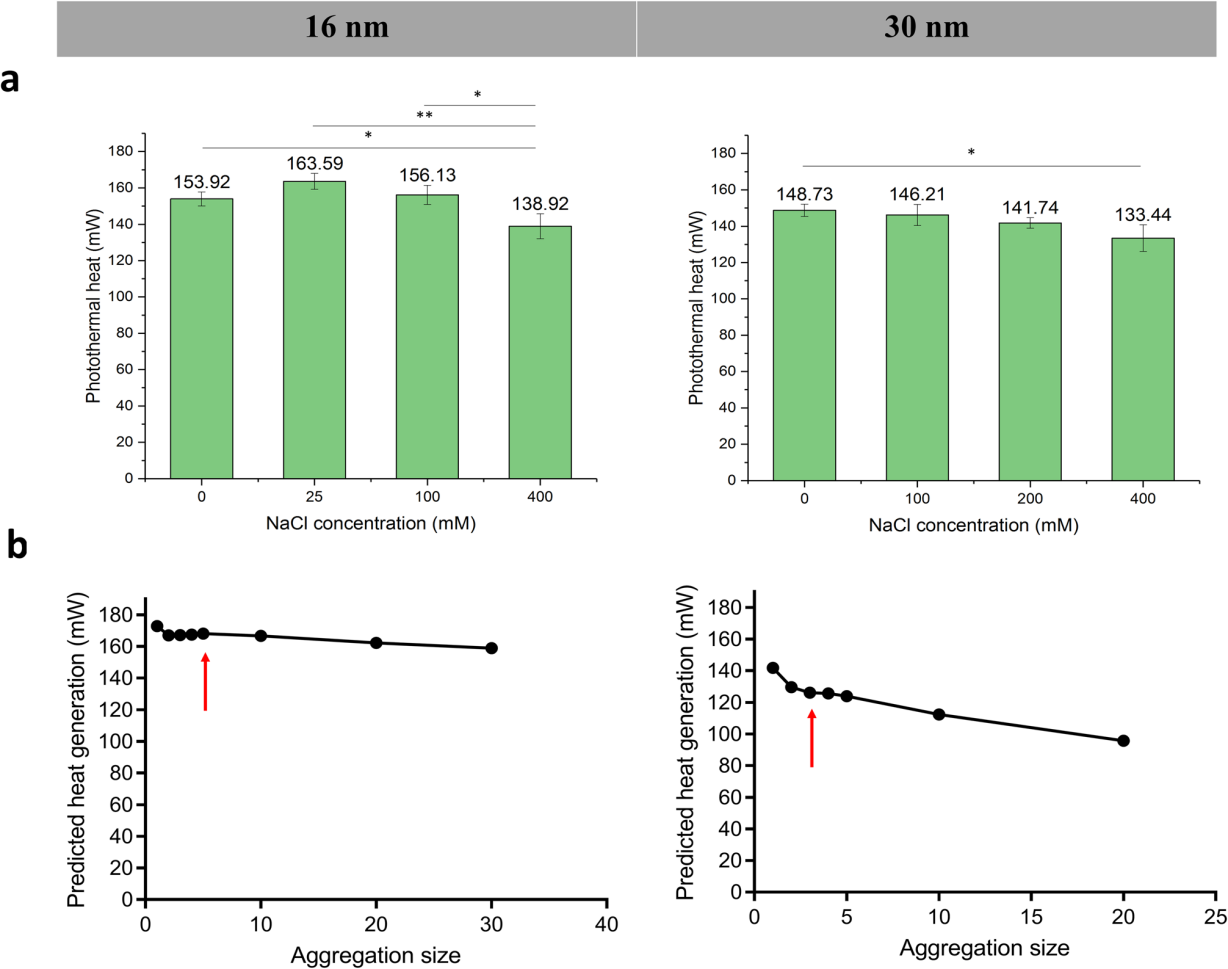
(**a**) Photothermal conversion experiment result of aggregated GNPs under 190 mW 532 nm wavelength CW laser. Large aggerates formed by 16 nm and 30 nm GNP showed significant lower heat comparing to discrete GNPs *p < 0.05, **p < 0.01. (**b**) Heat generation by converting optical properties predicted in modelling. Heating is shown to decline consistently with larger size aggregates. Predicted heat pointed by red arrows correspond to experimental heat generated by GNS aggregate with 400 mM NaCl addition.

For a better understanding the photothermal change, we measured their photothermal heating outcomes with the calibrated laser calorimetry approach as pictured in Fig. 3a. Equivalent volume and concentration solutions with different aggregation conditions of 16 and 30 nm GNS were heated by the same power 532 nm laser until steady state and photothermal heat conversion was extracted and shown in Fig. 4a. Although the average heating increased at small aggregation sizes (2 GNS) in 16 nm GNS aggregates, the increase was not significant (p = 0.1827). Both 16 nm and 30 nm GNS showed significant heating decrease (10%) in the largest aggregates induced by 400 mM NaCl addition (p < 0.05).

The salt-induced aggregation and stabilization method for controlled aggregation proved to be thermally stable during heating. This was achieved by fixing the aggregates prior to laser heating with either transferrin or PVP. The absorption spectrum of all the solution samples tested in this study showed no noticeable change one week after preparation or after a heating experiment.

### Comparison of calculation and experimental results

Both numerical modelling of optical properties and experimental heating of GNS aggregated GNS suggest a general but modest reduction in heating capabilities of GNS in the aggregated state. To provide a more direct comparison between the modelling and experimental results, the calculated optical properties of 16 nm, 30 nm GNS excited by 523 nm laser were converted to heating and shown in Fig. 4b. Both 16 nm and 30 nm GNS showed declined heat generation with more aggregation. Based on DLS results, 400 mM NaCl induced 5-GNS aggregates with 16 nm GNS and 3-GNS aggregates with 30 nm GNS in average. Our experimental data matched with the predicted trend, where large GNS aggregates (induced by 400 mM NaCl) showed significant lower photothermal heating (p < 0.05) comparing to monomeric GNS (as made) without NaCl addition. It is noted that the absolute values were not matched between the prediction and experiments. This is because the NaCl induced aggregates are not uniform in size and shape (Fig. 3b,c). Moreover, experimental GNS aggregates cannot be expected to be as densely packed as assumed in modelling. This was by intention as it is computationally expensive study and therefore, we chose to focus on an extreme case of compact aggregate geometries only. For GNS aggregation in biosystems, the shape of aggregation clusters could be irregular, and the particles could be gapped (i.e. with no direct contact). To understand how aggregation geometry affected the optical properties, we also generated some sparse geometries and performed DDA on them (Table S1). Our results show that compact aggregates heat better than sparse ones in all GNS and aggregate cluster sizes at the extinction peak (Fig. S5). This could be explained by the more randomized dipole coupling in compact geometries in comparison to the relatively more linear-shaped sparse geometries. On the other hand, the gap distance effect has been studied on GNS dimers in previous studies which found that the primary peak amplitude increases with gap distance, or in other words the optical properties of loosely aggregated GNS became closer to an isolated GNS spectra^23^. Therefore, aggregate structure and interparticle gap distance exert opposite influences on photothermal heating of GNS aggregates within the geometries we studied. With the stable, repeatable GNS aggregate samples synthesized, we were able to experimentally compare the photothermal conversion of different aggregation conditions.

Nanoparticle aggregation in-vivo is ubiquitous due to high salt concentration and interaction between nanoparticle and proteins regardless of nanoparticle type and size^16,46^. The physiological range of NaCl concentration in most biological fluids is 150 mM^47,48^. Herein, we physically tested 16 nm and 30 nm GNS aggregates induced by NaCl with a concentration in the range of 25–400 mM and stabilized the aggregates with stabilizers. For the range of GNS sizes, and aggregation sizes we tested, aggregation does reduce the photothermal heating up to 10% experimentally. It is worth mentioning that without stabilization, aggregate size will keep increasing and further decrease in GNS photothermal heating would be expected. Moreover, optical extinction has been measured with increasing of aggregate size by other groups^40,43^. Thus, we suggest the assumption of 17–28% reduction in heating during conditions that favor aggregation vs. idealized non-aggregated condition GNS based on our calculation results.

Our study also suggests that aggregation affects GNS photothermal heating in a complicated way that may inspire the design of new GNS and/or GNS photothermal systems. For instance, keeping the amount of gold constant, a large single GNS will heat more efficiently than a cluster of smaller GNS. Also, isolating individual GNS will likely improve the photothermal heating (e.g. adding a silica or other coating layer) by increasing the inter-particle distance and avoiding aggregation as dimers and trimers are experimentally difficult to avoid. This may be particularly important for GNS of more complicated geometries, such as GNR. We emphasize that careful reporting of experimental photothermal measurements of nano-heaters is necessary since our results show that heating is highly affected by the aggregation and distribution states (i.e. polydispersity and aggregation). Finally, this work provides a framework for quantitative characterization of photothermal conversion assessment in GNS and other simple or complex nanostructures. Future work will translate this work to the study of GNS photothermal heating in biological systems where aggregation is expected to play a role (e.g. cultured cells, embryos and tumors).

## Conclusions

This work reports a theoretical and experimental study of photothermal conversion of GNS over a wide range of size and aggregation conditions using DDA, UV–Vis and laser calorimetry. DDA modelling showed that aggregation reduces the per-particle extinction cross section compared to a discrete GNS at 532 nm excitation wavelength. Aggregates (≤ 30 GNS) formed by smaller GNS (5 and 16 nm whose resonance peaks are around 550 nm) showed a higher extinction at 808 nm, while the extinction of 30 nm GNS aggregates decrease at 808 nm excitation. Experimental work using a 523 nm CW laser was supportive, showing that aggregates from 16 nm (≥ 5 GNS) and 30 nm GNS (≥ 3 GNS) heat significantly less than dispersed discrete GNS. Together modelling and experiments show that laser heating in aggregated GNS systems shows a general reduction in heating on an average per-particle basis at 532 nm excitation. Our data show that this effect depends on GNS sizes, aggregation sizes, and geometry (dense vs. sparse). The general framework we provide here could lead to further studies, in which nanoparticle aggregation impedes laser heating and appropriate measures should be taken to obtain optimal experimental outcomes in specific applications.

## Supplementary Information

Supplementary Information.
