# Supplementary material for: Aggregation affects optical properties and photothermal heating of gold nanospheres
Source: Sci Rep. 2021 Jan 13;11:898. doi: 10.1038/s41598-020-79393-w (PMC7806971; doi:10.1038/s41598-020-79393-w)
Supplement: Supplementary file 1 — Supplementary Information. [file 41598_2020_79393_MOESM1_ESM.pdf]

Supporting Information

Aggregation Affects Optical Properties and Photothermal Heating of Gold Nanospheres

Yiru Wang[a,1], Zhe Gao[a,1], Zonghu Han[a], Yilin Liu[a], Huan Yang[a], Taner Akkin[b], Christopher J. Hogan Jr[a], and John C. Bischof[*a,b]

[a] Department of Mechanical Engineering, University of Minnesota – Twin Cities, Minneapolis, MN 55455, US

[b] Department of Biomedical Engineering, University of Minnesota – Twin Cities, Minneapolis, MN 55455, US

[1] These authors contributed equally to this work

*To whom correspondence should be addressed: bischof@umn.edu

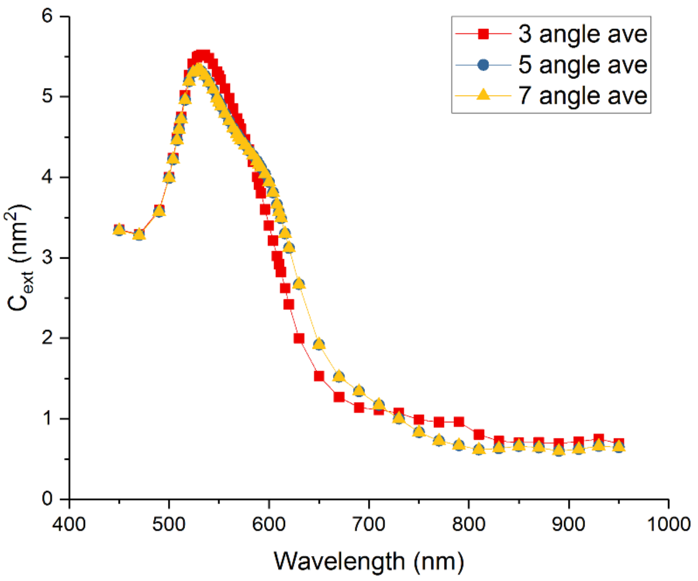

Figure S1. Directional independence verification of DDA configuration. Results of 3, 5, and 7 different incident angles were averaged for 5 nm GNS in three particle compact aggregation geometry. 5 was chosen as the number of averaged angles parameter in our DDA cases.

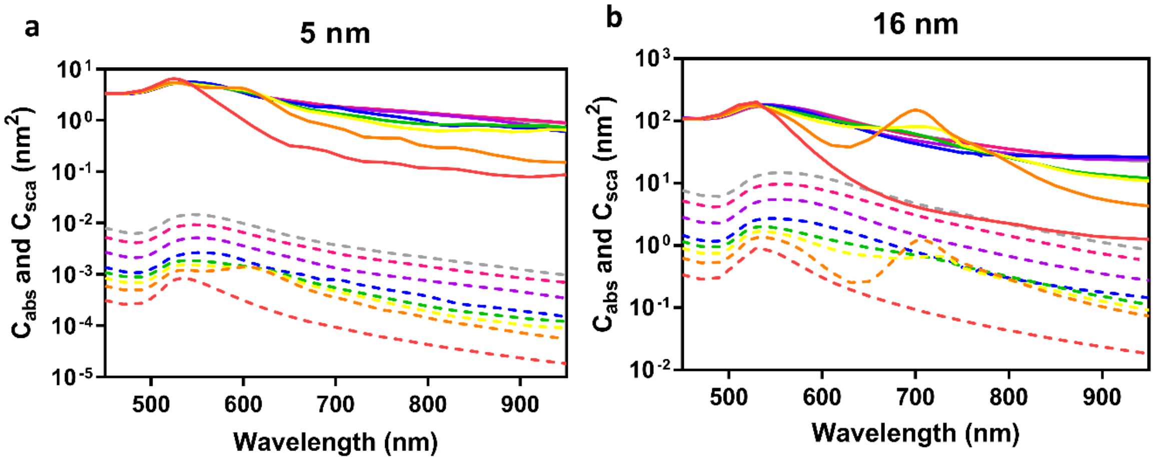

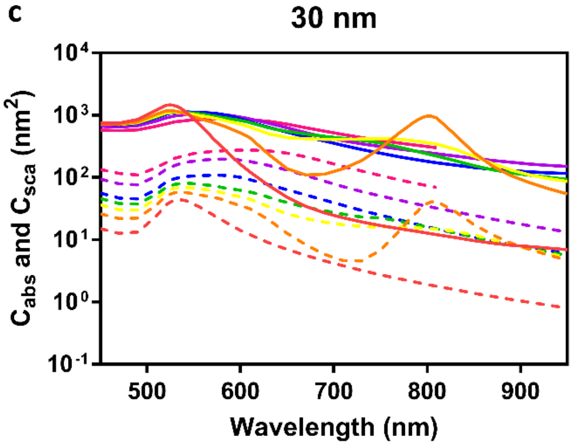

Symbol/color	Meaning
Solid line	C_{abs}
Dash line	C_{sca}
Red	1 particle
Orange	2 aggregates
Yellow	3 aggregates
Green	4 aggregates
Blue	5 aggregates
Purple	10 aggregates
Pink	20 aggregates
Gray	30 aggregates

Figure S2. Comparison between absorption and scattering cross sections in dense geometries.

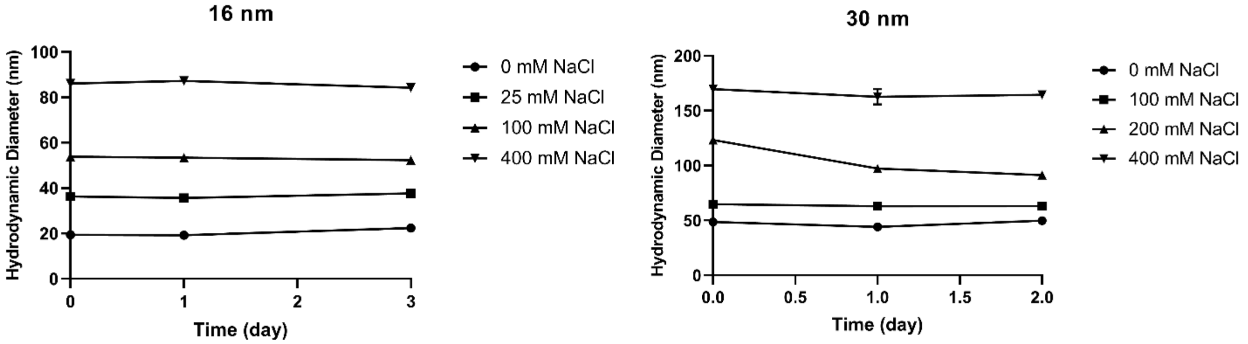

Figure S3. Hydrodynamic diameter of GNS and stablized GNS aggregates over time.

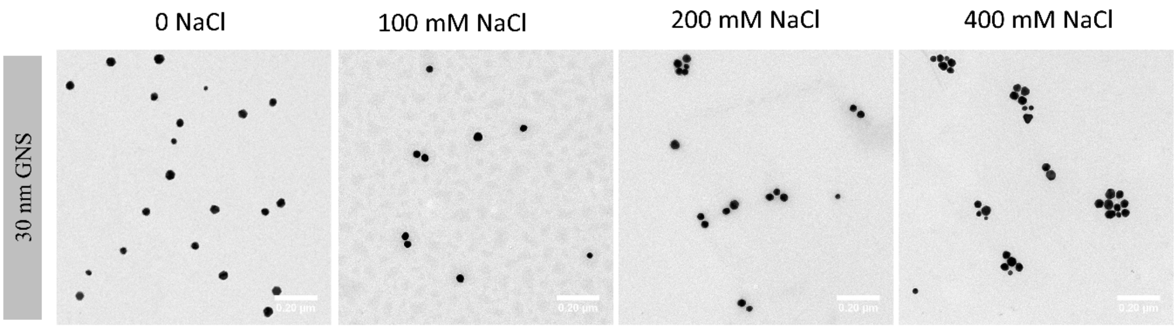

Figure S4. Additional zoomed-out TEM pictures of 30 nm diameter GNS aggregates. Scale bar is 200 nm.

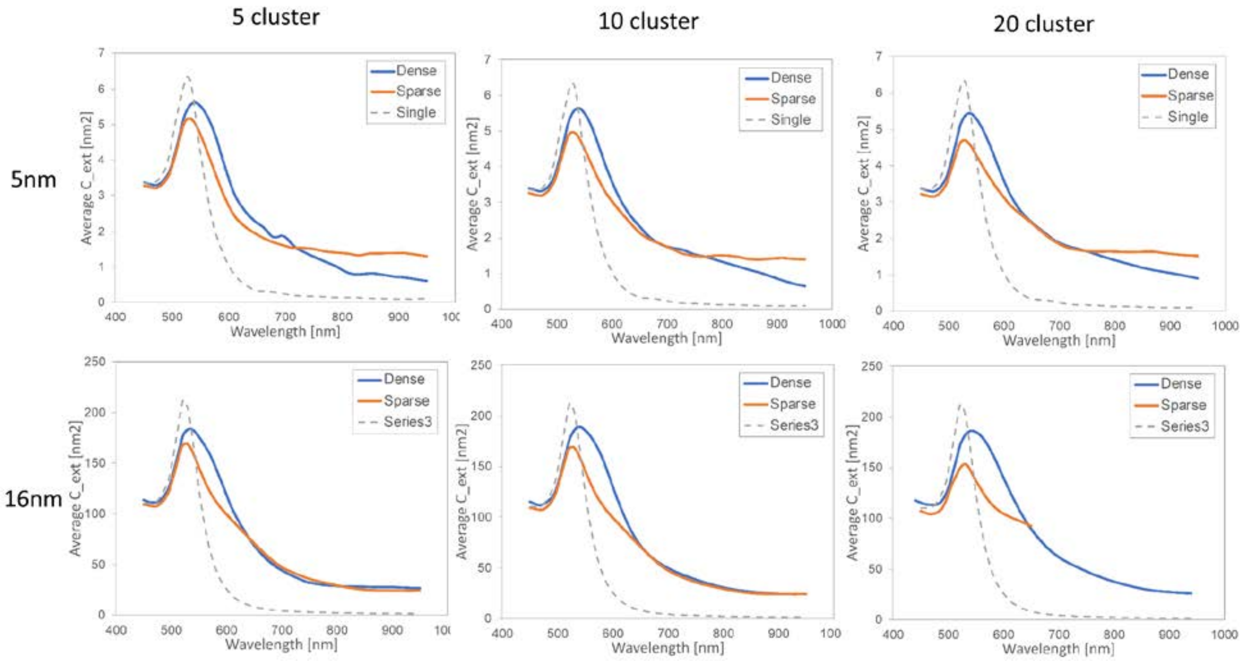

Figure S5. Average extinction spectra of dense and sparse aggregation geometries.

Table S1 Sparse GNS aggregate geometries

**Size**	5 GNS	10 GNS	20 GNS
**Geometry** (Sparse)	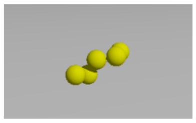	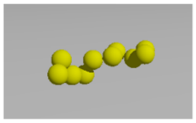	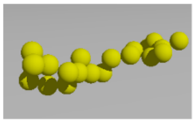
